# figManagement of Renal Vascular Injuries by Angioembolization in a Low Resource Setting: A Case Series with Review of Literature

**DOI:** 10.15388/Amed.2024.31.2.24

**Published:** 2024-12-04

**Authors:** Saifullah Khalid, Manzoor Ahmad, Ezaz Ahmed, Wasif Mohammad, AliImad Ali

**Affiliations:** 1 Department of Radiodiagnosis, JNMCH, AMU, Aligarh, Uttar Pradesh, India; 2 Department of Surgery, JNMCH, AMU, Aligarh, Uttar Pradesh, India

**Keywords:** renal vascular injury, angioembolization, diagnostic catheter, low resource setting, Raktažodžiai: inkstų kraujagyslių pažeidimas, angioembolizacija, diagnostinis kateteris, mažų išteklių aplinka

## Abstract

**Background:**

Renal vascular injuries, like renal artery pseudoaneurysms and arteriovenous fistulas, are rare but serious complications following renal procedures and trauma. Standard management requires costly microcatheters for angioembolization. This study investigates a cost-effective method using diagnostic catheters and affordable coils in a low-resource setting.

**Methods:**

We analyzed five patients from December 2023 to June 2024 who had persistent hematuria or hemoglobin drops after renal intervention or trauma. CT angiography identified their injuries, and angioembolization was performed with diagnostic catheters and coils, reducing procedural costs.

**Results:**

The use of diagnostic catheters and coils successfully managed all cases. Patients experienced resolution of hematuria and were discharged in 2–3 days. Follow-up over three months revealed no recurrence or significant renal function issues.

**Conclusion:**

Diagnostic catheters and coils offer an effective method for managing renal vascular injuries in resource-limited settings, achieving satisfactory outcomes with minimal morbidity.

## Abstract (it)

**Įvadas:**

Inkstų kraujagyslių pažeidimai, tokie kaip inkstų arterijos pseudoaneurizmos ir arterioveninės fistulės, yra retos, bet rimtos komplikacijos po inkstų procedūrų ir traumų. Standartiniam gydymui reikia brangių mikrokateterių angioembolizacijai. Šiame tyrime nagrinėjamas ekonomiškai efektyvus metodas naudojant diagnostinius kateterius ir prieinamas spirales mažai išteklių turinčioje aplinkoje.

**Metodai:**

Nuo 2023 m. gruodžio mėn. iki 2024 m. birželio mėn. stebėjome penkis pacientus, kuriems po inkstų intervencijos ar traumos išliko hematurija ar sumažėjo hemoglobino kiekis. Kompiuterinės tomografijos angiografija nustatė jų sužalojimus, o angioembolizacija buvo atlikta diagnostiniais kateteriais ir ritėmis, taip sumažinant procedūros išlaidas.

**Rezultatai:**

Visais atvejais sėkmingai buvo naudojami diagnostiniai kateteriai ir spiralės. Pacientams išnyko hematurija ir jie buvo išrašyti per 2–3 dienas. Tris mėnesius trukusio stebėjimo metu nenustatyta nei ligos pasikartojimo, nei reikšmingų inkstų funkcijos sutrikimų.

**Išvados:**

Diagnostiniai kateteriai ir ritės yra veiksmingas būdas gydyti inkstų kraujagyslių pažeidimus ribotų išteklių aplinkoje, pasiekiama patenkinamų rezultatų ir minimalaus sergamumo.

## Introduction

Renal vascular injuries like renal artery pseudoaneurysm and arteriovenous fistula are rare but clinically significant and a life threatening complications following surgical intervention and trauma. [1] The incidence of renal artery pseudoaneurysm after intervention as reported in current literature is low (0.6–1%). [2] However, some patients may present with persistent hematuria, flank pain and sometimes with hemodynamic instability requiring blood transfusion. [3] In postoperative patients or patients with a history of trauma, a high index of suspicion is necessary if they present with the above complaints. Moreover, appropriate imaging is also required to identify the source of bleeding and treat the vascular injury. Renal arteriography has been successful in identifying these lesions with adequate satisfaction. [3] Angiographic management, generally by superselective angioembolization of the injured vessel has success rates that exceed 95% and has replaced the need for open surgery. [2,4]

Renal artery embolization (RAE) was first described by Almgard in 1973. [4] RAE is defined as the voluntary occlusion of the renal artery or one or more of its branches, by means of temporary or definitive agents introduced through endo-vascular catheter.[5] In the standard procedure, a coaxial microcatheter is advanced which allows superselective access to the feeding vessel and further embolization of the pseudoaneurysm using coils or glue. [6]

As the microcatheters are expensive it becomes difficult to procure them by patients belonging to low socioeconomic status. So, this paper demonstrates our experience in using diagnostic catheter and 0.035” coils which are cheaper, thus decreasing the procedural cost in the management of renal vascular injuries.

## Patients and Methods

Patients who underwent an open or percutaneous renal procedure or trauma to kidney were included in the study if they had persistent visible hematuria or drop in haemoglobin but were hemodynamically stable. Initially a conservative approach was adapted but the hematuria did not resolve. They were then subjected to Computed Tomography (CT) angiography and were found to be having either a pseudoaneurysm or an arteriovenous (AV) fistula in one of the peripheral segmental arteries of the kidney. A total of five cases were included in this study from December 2023 to June 2024 (Table 1).

**Table 1:** Presentation of five cases and their management of renal vascular injuries

Cases	Age/Sex	Clinical Details	Angiography	Hardware used	Clinical follow up	Creatinine Values
1 (Fig 3)	24Y/M	History of Right PCNL for nephrolithiasis with complaint of blood in urine and increasing difficulty in passing urine five days after the procedure.	A pseudoanerysm arising from the lower segmental right renal artery measuring 3x3mm with DJ stent in situ.	A coil of size 2’’x2’’ was deployed in the polar artery at the lower pole through a COBRA 2 MERIT OEM diagnostic catheter.	Followed up for one year in Urology and IR OPDs. No recurrence of symptoms, Hemoglobin and Creatinine stable.	Pre: 1.5mg/dl Post: 0.6mg/dl
2 (Fig 4)	25Y/M	History of Left PCNL with DJ stenting presented with complaints of fever and chills with visible hematuria and urinary retention a week after the procedure.	Two pseudo aneurysms arising from lower segmental branch of left main renal artery measuring 16x11mm and 3x2mm with moderate left hydronephrosis with hematoma within the left pelvicalyceal system with hypo functioning of the left kidney.	Diagnostic catheter was advanced to the feeding vessel and two coils of sizes 2’’x5’’ and 2”x2” were deployed using Cook Medical Torcon BN Advantage Catheter ROC 5Fx65cm.	Followed up for six months in Urology and IR OPDs. No recurrence of symptoms, Hemoglobin and Creatinine stable.	Pre: 2.1mg/dl Post: 1.0mg/dl
3 (Fig 5)	55Y/M	History of open pyelolithotomy with complaints of gross hematuria five days after the procedure. On examination, pallor was visible and tachycardia was present. Hemoglobin was 6.9 g/dl.	A pseudo aneurysm arising from the interlobar branches of right main renal artery at the lower pole of right kidney measuring 15x10mm.	A coil of size 2’’x4’’ was deployed in the polar artery at the lower pole through a diagnostic catheter Cook Medical Torcon NB Advantage Catheter C2 5Fx150cm.	Followed up for six months in Urology and IR OPDs. No recurrence of symptoms, Hemoglobin and Creatinine stable.	Pre: 1.8mg/dl Post: 0.5mg/dl
4 (Fig 6)	18Y/M	History of blunt trauma to abdomen presented with visible hematuria two days after the injury.	Grade 4 Left Renal Injury with active excretion of contrast from the kidney. An arteriovenous(AV) fistula was seen as out pouching in upper pole of left kidney.	While attempting to navigate a sharp bend, as shown in the image below, the artery distal to the bend went into dissection leading to an occlusion of the fistula using Cook Medical Torcon NB Advantage Catheter C2 5Fx150cm.	Followed up for three months in Urology and IR OPDs. No recurrence of symptoms, Hemoglobin and Creatinine stable.	Pre: 1.9mg/dl Post: 0.7mg/dl
5 (Fig 7)	48Y/F	History of left PCNL with complaint of visible hematuria and persistent heaviness in the left flank six days after the procedure.	A pseudo aneurysm involving the left lower lobar renal artery measuring 10x11mm and an associated arteriovenous fistula.	Diagnostic catheter was advanced to the feeding vessel and two coils of sizes 2’’x5’’ and 2”x2” were deployed using COBRA 2 MERIT OEM diagnostic catheter.	Followed up for three months in Urology and IR OPDs. No recurrence of symptoms, Hemoglobin and Creatinine stable.	Pre: 1.4mg/dl Post: 0.5mg/dl

These patients were subsequently planned for angioembolization. Complete blood count and coagulation parameters were done preoperatively and packed red cell transfusion was done if needed. Informed consent was taken from the patient for the procedure and research purpose.

**Fig 1:**
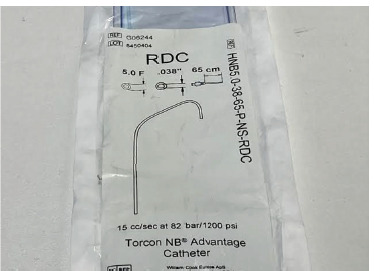
Micro Catheter

**Fig 2:**
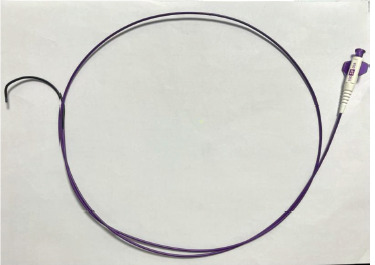
Diagnostic Catheter

The conventional technique is use of microcatheters (Fig. 1) to gain access into the vessel that has developed a pseudoaneurysm or an AV fistula. The microcatheter is usually obtained at a cost of $240 a piece and usually one to two catheters are required for the procedure which makes the cost of the total procedure around $550. In our centre, we used diagnostic catheters (Fig. 2) which greatly reduced this cost to within $150.

**Fig 3:**
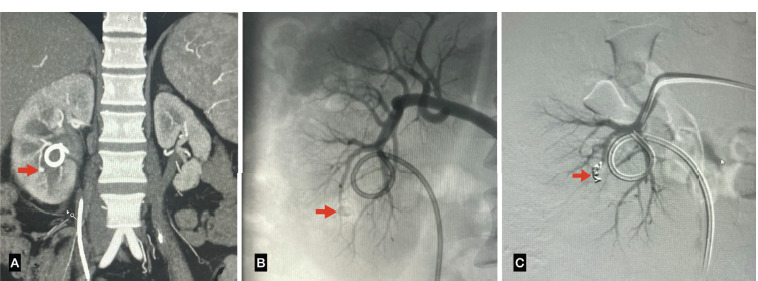
(A) CT Angiography showing a pseudo aneurysm in right lower segmental renal artery. (B) Diagnostic angiogram showing a pseudo aneurysm in the right lower renal artery. (C) Post embolisation showing a coil in the lobar artery causing causing occlusion of the pseudo aneyrysm.

**Fig 4:**
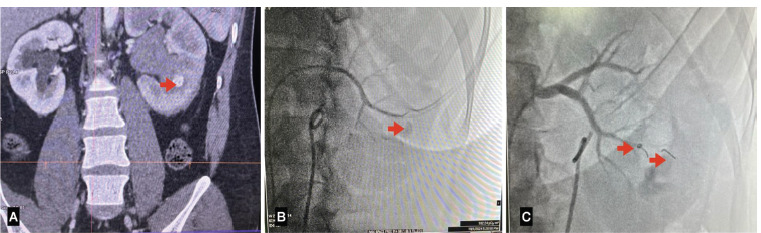
(A) CT Angiography image showing pseudo aneurysm in lower segmental left renal artery. (B) Diagnostic angiography showing two pseudoaneurysm in lower segmental renal artery of left kidney. (C) Post embolisation showing two coils in situ leading to occlusion of both pseudo aneyrysm.

**Fig 5:**
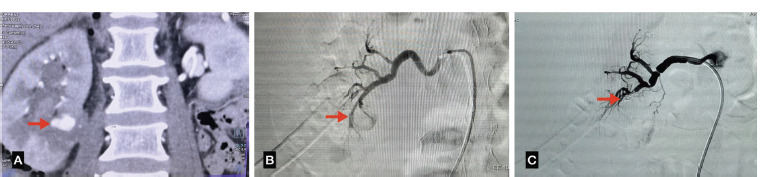
(A) CT Angiography showing a pseudo aneurysm in the right lower interloper renal artery. (B) Diagnostic angiogram showing a pseudo aneurysm in the right lower polar artery. (C) Post embolisation image showing a coil in the lobar artery causing occlusion of the pseudo aneurysm.

**Fig 6:**
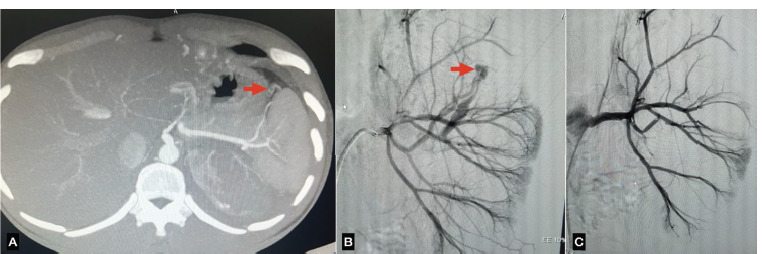
(A) CT Angiography showing an arteriovenous fistula in the right renal upper polar artery. (B) Diagnostic angiogram showing an arteriovenous fistula. (C) Post procedure angiographic image showing no evidence of AV fistula.

**Fig 7:**
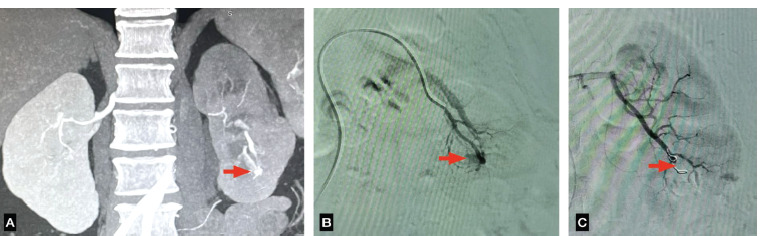
(A) CT Angiography showing an arteriovenous fistula in the lower polar left renal artery with an associated pseudo aneurysm. (B) Diagnostic angiogram showing an arteriovenous fistula and a pseudo aneurysm in the left lower polar artery. (C) Post embolisation showing two coils in situ leading to occlusion of both the pseudo aneurysm an AV fistula.

After obtaining access through the right femoral artery, check angiography was done which showed either pseudoaneurysm or AV fistula. Diagnostic catheter was then advanced to the feeding vessel and coils were deployed as needed to occlude the aneurysm or AV fistula. Subsequent check angiography done confirmed the closure of the pseudoaneurysm or the fistula. In one of the cases of an AV fistula, controlled arterial dissection was induced by means of the diagnostic catheter which led to closure of the fistula.

After intervention, hematuria significantly decreased and urine became clear in all the patients. The patients were discharged satisfactorily in the next 2–3 days under stable conditions. They were followed up in the Urology and IR clinic for the next three months. None of the patients had complaint of hematuria, they were vitally stage on follow up and no significant derangements were found in the renal function.

## Discussion

Percutaneous access to the upper urinary tract was first described in 1955 and PCNL was introduced somewhere around 1975. [7] Since then, many modifications have been made in the technique and presently, PCNL is considered to be the method of choice for clearance of large or complex renal stones. [7]

**Michel MS, Trojan L, Rassweiler JJ** reported that despite being a minimally invasive technique, PCNL can be associated with clinically significant bleeding. Transfusion rates in recent literature ranging between 5–18%. [8] In our case series, one out of the five patients had significant fall in haemoglobin and required two packed red cell transfusions.

**Srivastava A, Singh KJ, Suri A et al**. reported that major vascular complication caused by vessel injury during surgical intervention are pseudoaneurysm and arteriovenous fistula. They present as delayed postoperative bleeding after a mean delay of eight days. [2] In our case series, all the patients presented with visible haematuria after a mean delay of six to eight days. **Srivastava et al**. identified that vascular injuries postintervention or trauma is usually located in the periphery arteries. [2] In our experience also, all the vascular injuries were located in the peripheral arteries.

**Massulo-Anguiar MF, Campos CM, Rodrigues-Netto N Jr** reported that the diagnosis of intrarenal pseudoaneurysm is challenging. Currently, renal angiography is considered the standard for diagnosis, nevertheless, other imaging modalities have also been used with varied success rates. [9] In our experience, CT angiography was the diagnostic modality of choice and was utilized to localize the vascular injury. **Philippou P et al**. found that superselective embolization is highly efficient with success rates exceeding 90%. Occlusion using a microcatheter is achieved with deployment of coils, stents or endovascular glue. [1] However, the use of microcatheters and multiple coils of large sizes is usually not affordable in resource limited settings and populations of low socioeconomic status.

In a study conducted by **Andrea C et al.**, the patients who underwent angioembolization were followed up at 3–6 months with an ultrasonography or CT angiography in order to confirm treatment success and to identify clinically silent complications.

In our study, the patients were followed up for one year to three months and were assessed by clinical symptoms and renal function tests. The patients were clinically stable and no derangements were found. We did not opt for radiological follow up as the clinical and biochemical parameters were sufficient for monitoring. The follow-up imaging would be associated with additional radiation exposure and would also increase the cost. Imaging follow-up can be reserved in cases of doubts.

The technique followed in this study allows the use of a diagnostic catheter which can be procured because of its low cost as well as easy availability. The coils are deployed in the feeding artery instead of the pseudoaneurysm itself to occlude the lesion. Although, a section of the renal cortex is infarcted due to the occlusion of the feeding vessel, it does not carry significant morbidity to the patient as seen in the follow-up of our patients. Hence, this technique may be used in resource limited intervention settings with the consideration of sacrifice of a segmental artery accounting for limited infarct in the renal cortex as seen in the follow-up with routine renal function tests.

## Conclusion

Renal vascular injury after intervention or trauma, though a rare but clinically significant complication needs a high index of suspicion for diagnosis. Renal angiography or CT Angiography can be used to localize the lesion. Use of cheaper diagnostic catheters can be an alternative to the costlier micro catheters in low resource settings.

## Limitations

One major limitation of this study might be stated that after procedure follow was not done by parenchymal study. To improve results, in further studies, renal parenchymal imaging may be done to authenticate the findings.
